# Summarizing US Wildlife Trade with an Eye Toward Assessing the Risk of Infectious Disease Introduction

**DOI:** 10.1007/s10393-017-1211-7

**Published:** 2017-02-07

**Authors:** K. M. Smith, C. Zambrana-Torrelio, A. White, M. Asmussen, C. Machalaba, S. Kennedy, K. Lopez, T. M. Wolf, P. Daszak, D. A. Travis, W. B. Karesh

**Affiliations:** 1 EcoHealth Alliance, 460 West 34th Street, New York, NY 10001 USA; 2 The Food System Institute, LLC and Veterinary Population Medicine Department, College of Veterinary Medicine, University of Minnesota, St. Paul, MN USA; 3 Veterinary Population Medicine Department, College of Veterinary Medicine, University of Minnesota, St. Paul, MN USA; 4 Centro de Ecología, Instituto Venezolano de Investigaciones Científicas, Caracas, 1020-A Venezuela

**Keywords:** wildlife trade, legal trade, illegal trade, disease, species

## Abstract

The aim of this study was to characterize the role of the USA in the global exchange of wildlife and describe high volume trade with an eye toward prioritizing health risk assessment questions for further analysis. Here we summarize nearly 14 years (2000–2013) of the most comprehensive data available (USFWS LEMIS system), involving 11 billion individual specimens and an additional 977 million kilograms of wildlife. The majority of shipments contained mammals (27%), while the majority of specimens imported were shells (57%) and tropical fish (25%). Most imports were facilitated by the aquatic and pet industry, resulting in one-third of all shipments containing live animals. The importer reported origin of wildlife was 77.7% wild-caught and 17.7% captive-reared. Indonesia was the leading exporter of legal shipments, while Mexico was the leading source reported for illegal shipments. At the specimen level, China was the leading exporter of legal and illegal wildlife imports. The number of annual declared shipments doubled during the period examined, illustrating continually increasing demand, which reinforces the need to scale up capacity for border inspections, risk management protocols and disease surveillance. Most regulatory oversight of wildlife trade is aimed at conservation, rather than prevention of disease introduction.

## Introduction and Purpose

Major drivers of human–animal contact allowing pathogen exchange include animal domestication for companionship and food production, anthropocentric alteration of the environment and the global movement of animals and goods. Approximately one-quarter of human deaths are caused by infectious disease and nearly 60% of infectious diseases are considered zoonotic (pathogens transmissible between animals and humans); most of these (>70%) are caused by pathogens of wildlife origin (Taylor et al. 2001; Jones et al. 2008; Drexler 2010). Whereas historically disease spillover events were likely to remain local, even undetected due to natural, cultural and geographic barriers, modern transportation allows emerging diseases to spread along various globally connected networks in a matter of days. In the past decade alone, we have witnessed several novel disease threats to global health, food security and economic stability as a result of one of these networks—the trade of live wild animals and/or their products (Karesh et al. 2007, 2012).

Anthropogenic movement and manipulation of domestic and wild animals, including globalized trade, were proposed as “the” biggest potential trigger drivers for disease emergence and spread since the advent of agriculture [WHO, Food and Agriculture Organization (FAO) and World Organisation for Animal Health (OIE) joint report 2004]. Lack of wildlife trade surveillance and proper systematic management of the data available represents a major gap to understand and determine high-risk pathways of potentially adverse organisms’ introduction. In order to properly assess this threat to the USA, we must (1) better understand the scope of the trade in terms of species, volume, condition and origin; (2) determine high-risk pathways of introduction for further assessment; and (3) understand the regulatory framework that exists to manage these threats.

The goal of this study is to characterize the wildlife trade entering the USA as a baseline for understanding the magnitude of the potential threat these activities may pose to the environment, animals and humans. Although reports exist in the literature, to our knowledge this is the broadest summary (in terms of time scale and detail) aimed at supporting risk assessments surrounding US wildlife trade importation.

### The Global Wildlife Trade

Wildlife trade is one of the largest and most complex commerce exchanges in the world. The legal global trade in wildlife and wildlife products involves the movement of billions of plants and animals comprising an economic value estimated at US $300 billion per annum (Ahlenius 2008; WWF/Dalberg 2012). The illegal aspect of wildlife trade is estimated to be a $5–20 billion-dollar industry, comparable to the international trade of narcotics and weapons (Wyler and Sheikh 2008; Haken 2011; WWF/Dalberg 2012). There are no adequate estimates of the full scale of wildlife traded throughout the world given its diversity, scope and partial underground existence. Uncertainty surrounding this issue is enhanced by lack of international data standards and varying commitments to data collection infrastructure within and between countries.

Fundamental terms such as “legal” and “illegal,” “formal” and “informal” may be subjective and based upon which regulations are applied and the context of the trade (e.g., national laws vary by country for trade in a particular species, certain species can be traded for particular purposes but not for others). In some cases, legal trade is well recorded by border officials while in other cases it is largely ignored. Confiscated illegal trade is often reported but undetected illegal shipments regularly go unrecorded. Legality of wildlife trade in most instances does not correlate with disease risk posed, as the majority of wildlife trade regulations (e.g., the Convention on International Trade in Endangered Species of Wild Fauna and Flora; CITES) are in place to conserve certain species or regulate economies rather than protect health.

Although wildlife trade is often lumped into a single entity, this enterprise is comprised of a multitude of products such as food, trophies, pets, fashion, medicine, artifacts and aphrodisiacs. Within each category exist a range of specialty market value chains that vary in motivating economics, cultural and societal origins, geographic source and destination, transportation type and route, trader and consumer identities, behavioral practices, species volume and condition, local and international legality. This results in vastly variable threats including loss of biodiversity, invasive alien species, food security and emergence of both high- and low-consequence pathogens. Thus, threats can only be quantified in response to specific questions (i.e., examining unique traits of specific market chains/pathways).

### Legal Trade

Timber and plants are estimated to comprise nearly 70% of the known (broadly defined) wildlife global trade value, leaving non-aquaculture fisheries products responsible for 28% and ornamental fish, mammals, herpetofauna and other species responsible for roughly 2% (US $5.27 billion) (Engler and Parry-Jones 2007; Ahlenius 2008).

The majority of *live* wild animal trade is comprised of aquatic animals and herpetofauna traded mainly for the pet industry. China and Southeast Asian countries are the top global exporters, while the USA and European Union (EU) are the top importing consumers (Altherr et al. 2011) of aquatic and herpetofauna wildlife. A portion of this trade is recorded by weight only, leaving the total number of individual animals involved unquantified. Approximately 187 million live fish are imported to the USA annually, 92% of which are freshwater taxa (Smith et al. 2008). Live turtles and frogs are also commonly imported as pets as well as food items. The USA imports on average 2280 tons of frog legs in addition to 2216 tons of live frogs for consumption each year (Altherr et al. 2011).

Birds and mammals are also highly represented among a myriad of known global trade routes for exotic pets (Bush et al. 2014). A review of this trade found it to be an expanding, yet fluid and dynamic industry with reasons for its growth including human population expansion, increasing affluence in South America and East Asia (resulting in a larger market for exotic pets), use of the internet and a broadening interface with wildlife habitat (Bush et al. 2014).

### Illegal Trade

Given global variability in laws and difficulty in distinguishing between legal and illegal transactions (e.g., false declarations of geographic origin, captive vs. wild-caught, misrepresentation of purpose of import or final destination), monitoring legality of wildlife trade is comparable in complexity to weapons trade. In the majority of instances, the legality of trade of wildlife at the international and national level is determined by authorities tasked with conservation rather than public or animal health protection. Specifically, unpermitted trade of CITES-listed species across international borders comprises the bulk of what is considered and/or reported as global illegal wildlife trade.

As with legal trade of wildlife, species are traded illegally as exotic pets, specialty foods, traditional medicines, trophies and fashion items. Drivers of this illicit trade vary from financial to cultural to relic.

Because the drivers and components of illegal wildlife trade are highly variable, the perpetrators do not fit any one category nor does their trade behavior follow a single pathway. Diverging networks include local village hunters, criminal groups engaged in drugs or terrorism, government officials and other economically driven sellers and consumers (Hayman and Brack 2002; Warchol et al. 2003; Wyler and Sheikh 2008; WWF/Dalberg 2012).

### Trade Data

CITES maintains a database of reported trade of CITES-listed species only. The database is managed by the United Nations Environment Programme–World Conservation Management Center (UNEP–WCMC) and currently holds 7 million records of trade involving 50,000 scientific names of taxa listed by CITES. Currently, more than 500,000 trade records are reported annually (http://www.cites.org/eng/resources/trade.shtml).

There are also trade data held by the United Nations Statistics Division Comtrade. These data are maintained in broader categories such as “live animal” or “reptile skins.” Although some of these data are more specific, species level detailed information is generally not available (Chan et al. 2015). The Comtrade data are self-reported by trading partners, and as a result, there are inconsistencies and may also be variable reporting even within the broader categories.

Additionally, there are data held by national governments that vary widely in their format and scope, and rely largely upon efforts of authorities given national laws and priorities. These data are often not available to the public but some summaries may be found in gray literature reports. All importers of wildlife to the USA are mandated to submit a 3–177 (www.fws.gov/le/pdf/3177_1.pdf) request to the US Fish and Wildlife Service (USFWS) which in turn records details of the imports into the Law Enforcement Management Information System (LEMIS) database. This database includes both CITES and non-CITES species considered to be wildlife per the USFWS definition (50 CFR 14.4). This database therefore holds records of all declared wildlife imports to the USA and, theoretically, details of illegal imports confiscated by authorities at US ports of entry. Although some wildlife species are regulated by other US agencies such as the United States Department of Agriculture (USDA), Centers for Disease Control and Prevention (CDC), and Food and Drug Administration (FDA), imports of wildlife as defined by USFWS are also tracked in LEMIS despite this overlap in jurisdiction. Therefore, the LEMIS system represents a comprehensive data source for incoming wildlife to the USA (with few exceptions such as bushmeat items that are not determined to be of CITES origin). LEMIS data are maintained by USFWS for 5 years.

## Methods

### Wildlife Trade Data Review

Since 2005, LEMIS data spanning January 1, 2000–August 6, 2013, have been collected, standardized, cleaned (e.g., misspellings) and entered into a propriety database structure curated by EcoHealth Alliance (EHA). The dataset, entitled “WILDb,” includes taxonomical, geographic and count information for each shipment that entered the USA and its territories, allowing for pathway analyses to be conducted on a global level for wildlife that entered the USA. The results presented here are a preliminary analysis of this comprehensive database of wildlife trade into the USA. The dataset continues to be periodically updated.

## Results

### Total Volume

**Figure 1:**
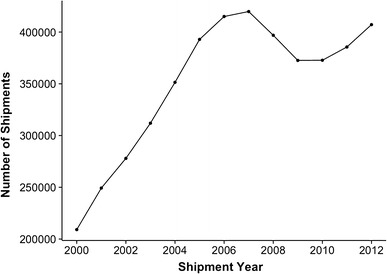
Trends of wildlife imports to the USA from 2000 to 2013 (note 2013 data are incomplete). The average annual number of shipments from 2000 to 2012 was 224,916,351 s: 42,377,484; median: 231,564,610.

As of January 2015, WILDb included a total of 5,207,420 individually identifiable wildlife shipments entering the USA between January 1, 2000, and August 6, 2013. The number of annually declared wildlife shipments doubled during the period examined (Figure 1), reaching approximately 400,000 declared shipments imported in 2012.

These shipments included a total of 11,033,468,322 individual specimens/animals, plus an additional 977,109,143 kg of specimens/animals measured only in weight. Of these, 3,028,647,093 (27.4%) individuals plus 24,449,892 (2.5%) kg were recorded as live upon entry. Thus, for the period 2000–2012, there was an annual average of 224.9 million (s = 42.3 million; median = 231.5 million) live animals plus an additional 1.8 million kilograms of live animals imported into the USA as recorded in the LEMIS database.

**Figure 2:**
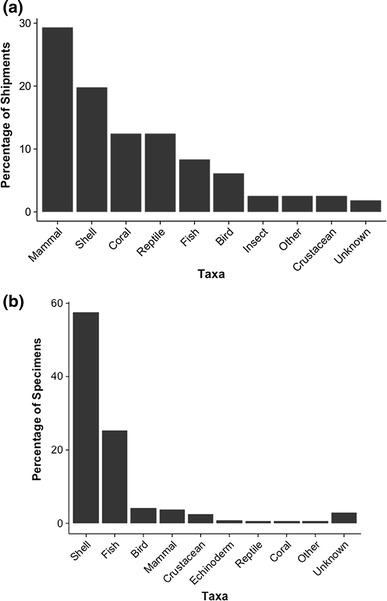
Relative percentage of taxa imported to the USA between 2000 and 2013, by **a** shipment, **b** specimen.

We selected the top ten categories represented by the data for illustration of the most frequent wildlife taxa imported to the USA. The majority of wildlife shipments (by taxon) contained mammal products (most of which were non-live; Figure 2a), while the majority of total specimens imported were shells and tropical fish (Figure 2b). This is due to the fact that shipments of mammals and their products contain fewer individuals or items while large volumes of aquatic species can be transported in a single shipment.

### Live Animals

**Figure 3:**
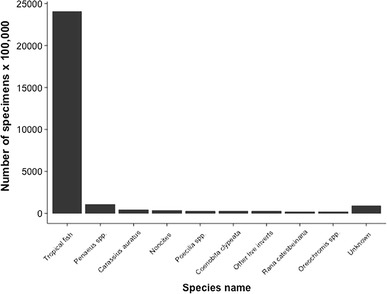
*Live* wildlife species (as species name or identity recorded in LEMIS) most frequently (top ten) imported to the USA between 2000 and 2013, by number of specimens (*Penaeus* spp.: prawn; *Carassius auratus*: goldfish; *Poecilia* spp.: molly and guppy fish; *Coenobita clypeatus*: Caribbean hermit crab; *Rana catesbeiana*: American bullfrog).

Nearly one-third of all wildlife shipments entering the USA contained live animals, the vast majority of which were imported by the aquatic and pet industry. Aquatic, amphibian and invertebrate species accounted for approximately 50% of recorded shipments of live animals to the USA. Top specimens involved in such live shipments are represented in Figure 3. Once the aforementioned subset is removed, reptile, rodent and bird species destined for the exotic pet trade make up the majority of remaining live imports. While roughly 27% of incoming live wildlife shipments contained mammals, this taxon only represented 4% of overall number of specimens imported (approximately 406,662,421 individual mammals, plus additional mammals documented only by weight vs. number of animals). Excluding those recorded only by weight, 2,434,851 live mammals were imported.

### Country of Origin

**Figure 4:**
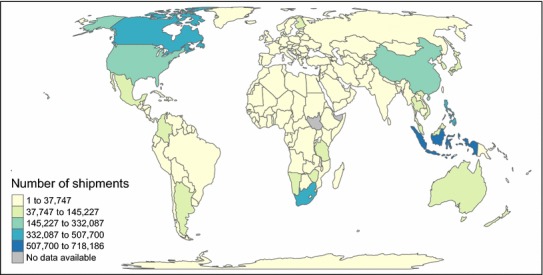
Map reflecting countries of origin of wildlife imports to the USA between 2000 and 2013, by shipment.

The data contained reported both “country of origin” and “country of export.” This reporting is by the importer/exporter, and therefore, country of origin may be falsely reported without means for authorities to verify source. The stated origin of imported *live* wildlife from 2000 to 2013 was roughly 77.7% wild and 17.7% captive (4.6% listed as ranched or other). Since many species traded may be wild-caught or captive-raised, it remains difficult for authorities to identify false reporting of wild versus captive and true country of origin, despite visual inspection and means of import. The reported countries of origin from 2000 to 2013 for all declared US wildlife imports by shipment are shown in Figure 4, with Indonesia as the leading exporter. However, at the specimen level (i.e., number of individual animals/products imported), China was the leading exporter. Many imports were not identified at the species level.

China and Southeast Asia was a primary region of origin for US wildlife imports. The vast majority of both live and non-live wildlife imported from this area were aquatic, invertebrate and herpetofauna species. Indonesia was responsible for exporting the most live wildlife shipments to the USA during the period examined, comprised mainly of these aforementioned species. However, live mammals and birds were also imported. Examples of identified live species imported from China included over 120,000 kg of live American bullfrogs (*Rana catesbiana*)—a conservatively estimated 360,000 frogs—imported mainly for food, nearly 30 million live pheasants (*Phasianus colchicus* spp.), approximately 150,000 live macaques, in addition to live bats for research and Asiatic chipmunks for pets in fewer numbers. Vietnam was the exporter of 300 shipments of live macaques to the USA during the period examined. Taiwan exported approximately 450,000 finch-like live pet birds including canaries and goldfinches, and Indonesia exported over 85,000 kg of “edible-nest swiftlet” nests to the USA.

The primary origins of mammal imports specifically were Canada and South Africa based on number of shipments, and the USA and China for number of specimens/animals imported. More than 15,000 live bison were imported from Canada annually, and additional amounts recorded only by weight. Likewise hoofstock made up the majority of mammal imports from South Africa. Wildlife imports that were listed as having the USA as country of origin included deer, squirrel, bear, alligator, avian products and aquatic species such as squid.

### Ports of Entry

**Figure 5:**
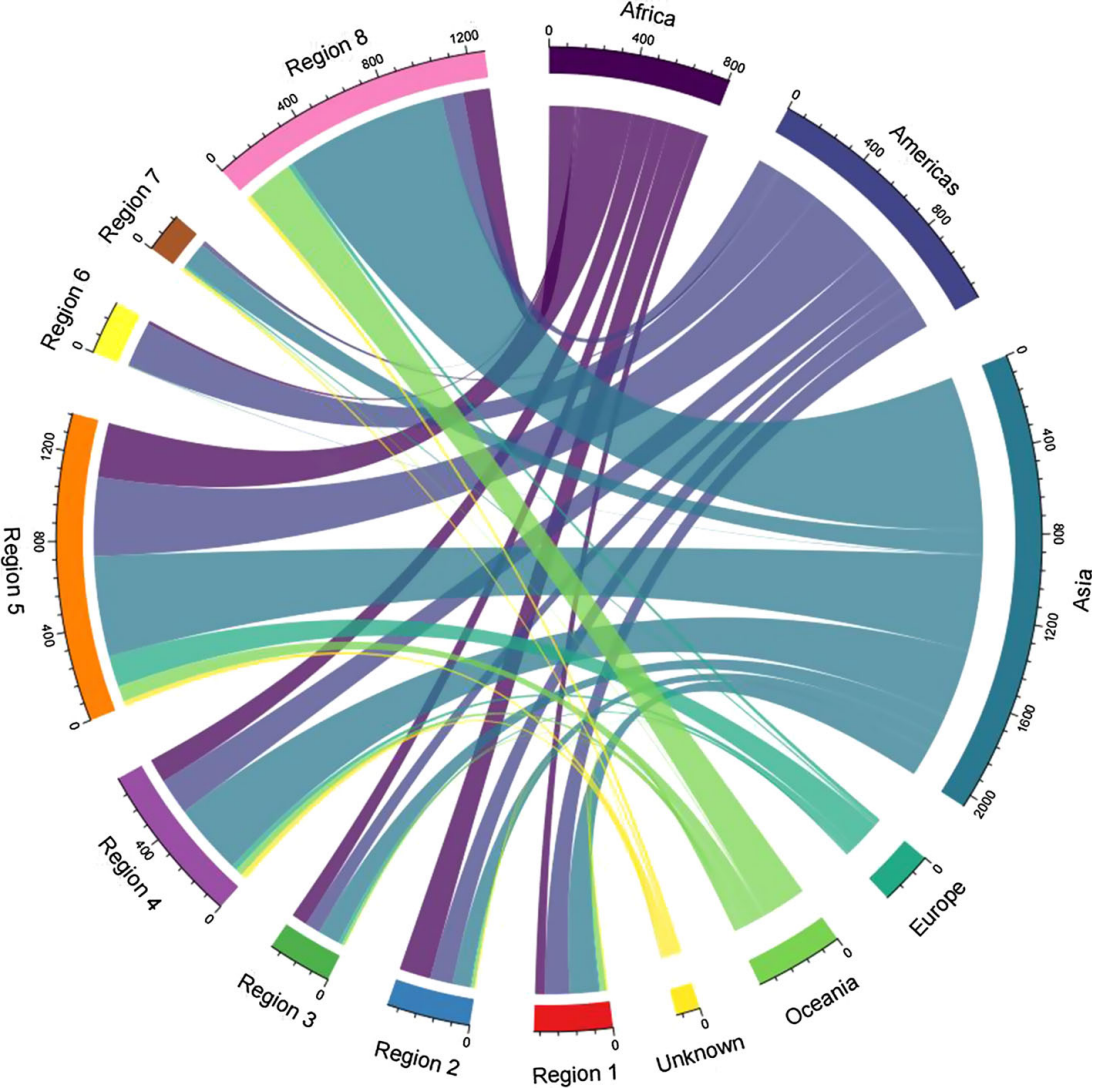
Circle plot representing the number of shipments (×1000) of wildlife from different continents of the world to US regional ports of entry between 2000 and 2013 (*Region 1*: Pacific; *Region 2*: southwest; *Region 3*: Great Lakes; *Region 4*: southeast; *Region 5*: northeast; *Region 6*: Mountain Prairie; *Region 7*: Alaska; *Region 8*: Pacific southwest).

Nearly half of all declared wildlife imports to the USA came through the ports of New York, Los Angeles and Miami (USFWS Regions 8, 5 and 4, respectively) (Figure 5).

### Refused Shipments

Ninety-nine percent of recorded imports were legally declared. This percentage is a reflection of the vast amount of declared trade recorded dwarfing the number of confiscations at US borders. Commonly refused imports (shipments deemed illegally imported to the USA by USFWS or other US agency regulations and thus refused entry) included sturgeon (caviar), baby harp seal pelts, Indian peafowl (peacock) feathers, white tailed deer products such as antler, elephant ivory (e.g., décor, trophies, jewelry), sea turtle products (e.g., leather), crocodilians (e.g., leather), musk deer (traditional medicine products), and reptile and ostrich products (e.g., leather).

**Figure 6:**
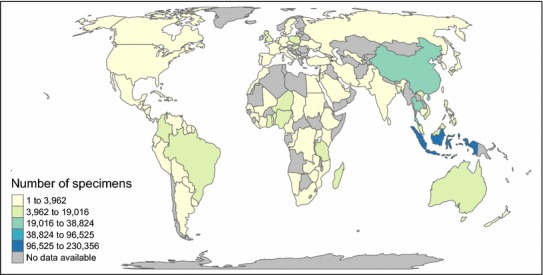
Map reflecting countries of origin of live refused wildlife imports to the USA between 2000 and 2013, by number of specimens. *Note* that China includes imports from Hong Kong as well.

While most illegal shipments presented at the Mexican border, the majority of illegal specimens (number of animals) that presented at ports of entry originated in China. Such items included deer and bear medicinal items, macaque scientific specimens, live aquatic species and reptiles. Documented origins of illegally imported live wildlife to the USA by specimen are illustrated in Figure 6, with Indonesia as the leading country of origin. The most common of these live refused specimens were comprised of corals, fish and herpetofauna from Southeast Asia, as well as birds and corals from the Caribbean. The most common origins of live, non-aquatic confiscations (by specimen) included herpetofauna and bird species from Africa, Asia and South America.

## Discussion

The USA is a top global consumer at the national level of legal wildlife and wildlife products according to records, along with China, and the EU as a whole (Asmussen et al., *unpubl. data*). To our knowledge, this is the most comprehensive report of US wildlife trade importation for this time period and of this scale. The most remarkable finding of this review is that the number of declared wildlife shipments into the USA has doubled since 2000. The economic value of wildlife imports paralleled this increase in shipments, rising 108% from 1998 to 2007 (Ferrier 2009).

### Pathway Analysis

#### Species

Over 11 billion specimens and an additional 977 million kilograms of wildlife were imported during the period examined, with one-third of shipments containing live animals, mostly for the aquatic and pet trade. With this volume of live wildlife entering the USA for commercial purposes, concerns have been raised regarding the unwanted side effect of invasive alien species and their pathogens. The 50,000 recorded invasive alien species imported to the USA have cost the government an estimated US $120 billion per year (Pimentel et al. 2005) in damage or control efforts. Over 200 species of imported fish have resulted in introductions to the wild in the USA with nearly half establishing breeding populations at least for some time (Smith et al. 2008). Beyond the environmental impacts, translocation of such live wildlife has resulted in pathogen pollution (the introduction of viruses, bacteria, fungi and parasites into new environments) with consequences to native wildlife health and US fisheries, for example (Springborn et al. 2015).

While in some cases wild-caught specimens are more likely to harbor pathogens due to previous exposure, poorly captive-reared species also may serve as a source of pathogens. It is suspected that there is a high frequency of false reporting regarding wild versus captive origin given that many species are legal to trade if captive-raised but not if wild-caught; many species are easier and cheaper to catch than to breed; and it is nearly impossible for officials to tell the difference between wild-caught or captive-reared specimens. This is the case in seafood trade where genetic testing data have identified many examples of mislabeling of species and origin (Warner et al. 2013).

It is not surprising that the vast majority of imports (by specimen number) consist of aquatic species and herpetofauna; given that these animals are often shipped together in large numbers, they are in high demand by consumers and a number of other factors (e.g., small size, lack of requirement for individual health certificates, less likelihood of being protected). The fact that these species are likely to be shipped live and in large numbers means that a significant number could survive even given long distance shipment from Asia. The survival of large numbers of highly stressed live animals entering the USA increases the overall risk of disease introduction.

#### Source Countries

The majority of individual specimens entering the USA, most of which were aquatic species, were from China and Southeast Asia. The degree to which such shipments pose a risk to US natural resources in terms of aquatic pathogen introduction is likely dependent upon their final destination and disposition, as well as how water used in shipments is disposed. Mammal and bird species were also imported from this region, with several notable imports such as swiftlet nests, over half of which were imported since the 2005 emergence of H5N1 highly pathogenic avian influenza (HPAI) (swiftlets were proven host species of avian influenza in Vietnam, although to our knowledge no nests have tested positive; FAO EMPRES/GLEWS, 25 April 2013).

Canada and South Africa were responsible for importing significant numbers of mammals, including live hoofstock and their products. Interestingly, the USA is a primary country of origin of its own imports. This may occur if an item passes through another country such as takes place when importing wildlife from Alaska to the lower 48 states via Canada or if wildlife species are exported for processing and then re-imported, as we do with some agricultural species.

The majority of illegal shipments of live non-aquatic wildlife were confiscated at the Mexican border, especially those containing reptiles and birds imported for the pet trade (Ferrier 2009). Overall, most non-aquatic confiscations were from African and Asian countries and were comprised largely of reptiles as well as birds targeted for the pet trade.

#### Point of Entry

Nearly half of all declared wildlife imports to the USA came through the ports of New York, Los Angeles and Miami, thus providing opportunities for targeted strengthening of monitoring and law enforcement efforts. The vast majority of wildlife imports through New York are commercial, and 97% of declared wildlife imports come via air cargo (US Fish and Wildlife Service 2005). Reasons for high traffic through New York include the fact that it is a fashion capital, the home of many scientific and educational facilities, and a top port of entry for tropical fish importers (United States Fish and Wildlife Service (USFWS) (2004). Accordingly, the top live imports to New York are medicinal leeches and fish, while top commodities include caviar, shell products, furs and skins (United States Fish and Wildlife Service (USFWS) (2004). Los Angeles is also a predominantly commercial port when it pertains to wildlife imports. Over 80% of imports arrived via air and most remaining imports via ocean cargo. Main imports include live aquatic species and reptiles as well as shell products, jewelry, eggs and skin/hair products (US Fish and Wildlife Service 2005). Miami, the largest port of entry from Central and South America, showed similar trends, receiving over 90% of its imports by air, comprised mainly of fish and reptile species.

#### Use and Legality

The purpose of international illegal wildlife trade varies by region. For example, in China illicit imports are primarily for exotic foods, traditional medicines and trophies; caviar, fashion and exotic pets are in demand in the EU; and exotic pets, souvenir items and hunting products comprise most illegal imports to the USA (Wyler and Sheikh 2008; Ferrier 2009; Karesh et al. 2012; Bush et al. 2014).

Given existing trade and travel routes, much of the trade that enters North America passes via flight patterns from Africa and Asia via the EU (Asmussen et al., *unpubl. data*), itself a significant global consumer of illegal wildlife. From 2003 to 2004, the EU executed over 7000 seizures including 3.5 million CITES-listed items (Engler and Parry-Jones 2007). The annual seizure rate in the USA is similar to this number based on our analyses.

A 2009 review of the US LEMIS database found that only 1% of all commercial wildlife shipments, and 0.4% by value, were refused entry by USFWS (Ferrier 2009). This finding is in alignment with our review of the WILDb database from 2000 to 2013. However, this percentage is based on assessment of refused shipments in the LEMIS database and therefore does not take into account smuggled shipments not detected; detected by Department of Homeland Security Customs and Border Protection (DHS CBP) but not reported to USFWS; detected by USFWS but not entered into LEMIS after the fact; or detected by CBP but reported only to another regulating agency of the same item such as the CDC, USDA or FDA. As previously noted, the USFWS LEMIS database is mainly a reflection of approved or rejected wildlife imports that are declared to USFWS by the importer and that non-declared (and thus illegal) imports that are successfully detected are done so through the DHS CBP. These confiscations should be reported to USFWS and entered into LEMIS; however, confiscation data housed in CBP databases are not readily available to the public in a significant level of detail for comparison. Thus, the amount of illegally imported wildlife is more than likely an underestimate.

### Study Limitations

As with all big data, there is uncertainty in this dataset. For example, non-CITES-listed species imports often lacked detail in several areas of the USFWS LEMIS database, suggesting such shipments were less scrutinized. Typically, “species” was recorded by USFWS using a four-letter “species code.” However, codes exist for several taxonomic levels (species, genus and more general “non-CITES” or “NA” descriptors), and a large portion of the data did not include species level identification. Further, codes often overlapped and/or several different codes were used to describe a single species. Currently, WILDb contains 14,074 unique species codes. EHA was able to ascertain some level of taxonomic information for over 98% of the data entries despite the fact that the majority of these did not provide specific species identification. This study was further limited to wildlife imports that were either accepted or rejected by authorities and did not include illegal shipments that evaded authorities as those go inherently unrecorded and unrecognized.

Given the sheer volume of live wildlife and wildlife product imports to the USA, and the fact that most refused shipments were due to CITES status and not based upon the risk of disease introduction, we believe it is prudent to further assess risk of pathogen introduction via wildlife trade. The current regulatory atmosphere for this goal is highly fragmented. The USFWS currently does not focus primarily on disease prevention, but on conservation status; the CDC currently focuses on specific health risks associated with non-human primates, African rodents and bats; and the USDA regulates non-domestic hoofstock, birds and few other specific mammals that originate in countries positive for reportable diseases. These species are regulated for specific diseases and thus may be approved entry if deemed safe.

## Conclusion

Many countries of origin for legal and illegal wildlife imports to the USA include “hotspots” of emerging and reemerging infectious and zoonotic pathogens (Jones et al. 2008; Smith et al. 2009) such as HPAI, Middle East respiratory syndrome (MERS) coronavirus, Nipah virus and *Brucella* ssp., as well as economically important livestock diseases. Introducing disease purposefully or accidentally need not utilize illegal trade since regulations concerned with pathogen introduction via trade are focused mainly on domestic species (regulated by CDC and USDA) and not enforced by the agency primarily monitoring wildlife trade into the USA (USFWS).

Since the majority of regulatory oversight of the wildlife trade is not specifically aimed at prevention of disease introduction, it remains a challenge to prioritize collection of the relevant information or risk mitigation measures. The Congressional Research Service notes that while the USA is involved in CITES, and contributes to the Coalition Against Wildlife Trafficking and Association of Southeast Asian Nations (ASEAN) wildlife law enforcement network, the USA “does not participate in international efforts to regulate international wildlife trade to prevent disease transmission or invasive species, as no such international organization currently exists” (Wyler and Sheikh 2008).

In 2014, President Obama issued the National Strategy for Combating Wildlife Trafficking to guide federal agencies in the global fight against wildlife trade. Yet even after the recent Ebola outbreaks in Africa, disease has not been a priority in this fight. The USA does adhere to the World Trade Organization’s (WTO) Sanitary and Phytosanitary (SPS) Agreement, which regulates the international trade in animals, animal products and plants, and is a member of the OIE, which sets international health standards for animals and animal products, recently including wildlife. In an attempt to support this effort, EHA recently worked with the OIE to develop a comprehensive list of proven wildlife hosts of OIE-listed diseases in order to inform member countries of the broad range of potential carriers of diseases of importance and to raise awareness surrounding potential wildlife trade health risks (Smith et al., *unpubl. data*).

We do not yet have a comprehensive picture of the scope and associated health risks posed by the international trade of wildlife. However, it is clear that the USA is a global leader in legal and illegal wildlife consumption. The demand for wild animals for use as companion animals/pets has been responsible for the majority of the live animal trade in the Western Hemisphere. This market involves billions of individual live animals, ranging from invertebrates and corals to non-human primates, originating from all over the globe. The demand for trophies, fashion, traditional medicines and exotic foods are some of the main drivers of the importation of wildlife products. The import process provides an opportunity to reinforce “critical control points” prior to entry through US borders. This is especially pertinent given that there is very limited traceability of wildlife species once entry has been gained into the USA.

The overarching goal of this work is to mitigate risk of pathogen introduction to US agriculture via wildlife trade. To accomplish this, we must first understand and characterize trade pathways as described herein. Given the large volume of imports, limited enforcement resources and lack of surveillance tools and infrastructure for many wildlife spp., the authors believe there is great opportunity for both regulated and non-regulated diseases of importance to public, agricultural or wildlife health to enter the USA. Thus, there should be an emphasis within the US Government and wildlife disease communities on filling gaps in the data for high priority pathways in order to better characterize risk. Specifically, threats posed by (1) large volumes of live aquatic species, (2) wild animal host species not currently regulated (e.g., some rodents) and (3) species closely related to domestic agriculture (e.g., hoofstock/camels) that may enter the USA for multiple purposes were prioritized by this working group for further assessment.
